# EHMTI-0278. Onabotulinum toxin a for hemicrania continua: a case series of 8 patients

**DOI:** 10.1186/1129-2377-15-S1-C45

**Published:** 2014-09-18

**Authors:** S Miller, F Correia, S Lagrata, M Matharu

**Affiliations:** 1 Headache, UCL Institute of Neurology, London, UK; 2 Neurology, Centro Hospitalar do Porto, Oporto, Portugal

## Introduction

Hemicrania Continua (HC) is a strictly unilateral continuous headache that is exquisitely responsive to Indometacin but some patients cannot tolerate this drug. Evidence for other therapies is limited, often limited to single case reports.

## Aim

Onabotulinum Toxin A (BoNT-A) is licensed for use in chronic migraine. Previous case reports on its use in HC are limited to two patients. We present outcome data on eight patients with HC treated with BoNT-A.

## Methods

Eight patients with HC received BoNT-A treatment as per the PRE-EMPT protocol for chronic migraine. Clinical data was collected pre and post-treatment. Headache load (defined as [severity (on the visual analogue score)] x [duration] x [frequency] of headaches) of each patient was calculated before and after treatment.

## Results

The median number of treatments was 2 (range 1-8) and the median number of BoNT-A units injected 165 (range 134-185). Six patients showed a headache load reduction of over 50%. Median improvement in headache load post-treatment was 88.5% (0-100%) with a significant difference in headache load post-treatment (p=0.018). The median duration of effect was 9.5 weeks (0-20 weeks). Five of six responders were previously taking Indometacin on a daily basis. All were able to switch to intermittent use of the drug.

## Conclusions

BoNT-A may be a useful treatment for HC where Indometacin is not tolerated and adds another potential option to the limited therapeutic arsenal currently available.

